# Trends in Body Fat, Body Mass Index and Physical Fitness Among Male and Female College Students 

**DOI:** 10.3390/nu2101075

**Published:** 2010-10-25

**Authors:** Peter Pribis, Carol A. Burtnack, Sonya O. McKenzie, Jerome Thayer

**Affiliations:** 1 Department of Nutrition and Wellness, Andrews University, 8475 University Boulevard - Marsh Hall 313, Berrien Springs, MI 49104, USA; Email: burtnack@andrews.edu (C.A.B.); mckenzis@andrews.edu (S.O.M.); 2 Center for Statistical Services, Andrews University, 4195 Administration Drive, Berrien Springs, MI 49104, USA; Email: thayerj@andrews.edu (J.T.)

**Keywords:** students, obesity, BMI, % body fat, physical fitness, VO_2max_ levels

## Abstract

There have been many publications in recent years reporting on the quantity of physical activity among college students using indirect indicators such as steps walked per day or time spent on physical activities. The purpose of this study was to describe the trends of physical fitness related to BMI and body fat among university students between 1996 and 2008. The results showed a significant decline in the average fitness levels measured as an estimation of VO_2max_ for male and female students (p < 0.001 for both sexes). The linear trend for BMI by years was not significant for both sexes (p for males = 0.772, p for females = 0.253). On average, in the last 13 years, % body fat was increasing 0.513%/year for males and 0.654%/year for females. There is a significant indirect correlation between the student’s VO_2max_ levels and % body fat, r = −0.489; p < 0.001 for males; and r = −0.416, p < 0.001 for females. Approximately 23.9% of the variance in the VO_2max_ levels in males and 17.3% in females can be explained by the variance in % body fat. The results support recent findings that physical fitness among college students is declining and body fatness is increasing.

## 1. Introduction

In the recent decade, a decline in physical activity among college students has been observed [1,2,3]. Regular physical activity is an important part of a healthy lifestyle. It is associated with decreased risk of heart disease [4], obesity [5], and cancer [6] and related to psychological well-being with lower levels of stress [7,8,9] and better cognitive functioning [10]. Recent studies indicate that almost half of the U.S. college student population does not participate in moderate or vigorous physical activity [11]. There is an alarming decline in physical activity among college students compared with those in high school [12]. Based on self-reported height and weight, approximately 35% of US college students are overweight or obese [13]. 

There have been several publications in recent years reporting on the quantity of physical activity performed by college students [3,11,14]. The primary purpose of our study is to describe changes in the levels of physical fitness measured as estimation of maximum uptake of oxygen during a graded exercise (VO_2max_) in the last 13 years in relationship to measurements of obesity (BMI, % body fat) in the male and female population. Our study was conducted at Andrews University, which is a private Seventh-day Adventist (SDA) campus. SDAs represent a unique population to study because of their emphasis on healthy lifestyle [15,16]. This religious group strongly recommends for its members to follow a healthy lifestyle defined as proper use of “air, sunlight, temperance, rest, exercise, proper diet, water, and trust.” [17]. 

## 2. Materials and Methods

This descriptive longitudinal study analyzed MicroFit tests data accumulated between years 1996 to 2008. Since MicroFit testing was part of mandatory curricula for all physical activity classes, informed consent forms were not collected. The study was approved by the Institutional Review Board of Andrews University (IRB Protocol #07-119). 

### 2.1. Participants

Overall, 5101 students took the MicroFit test which was always done during the fall semester (September–December) and spring semester (January–April) of each year. Every Andrews University student that participated in a physical activity class was required to evaluate his/her physical fitness by taking the MicroFit test. 

### 2.2. MicroFit testing

MicroFit is a Food and Drug Administration (FDA) approved medical assessment tool which measures several parameters of physical fitness: body composition (weight, height and % body fat), blood pressure (systolic and diastolic), muscular strength, flexibility, resting heart rate, aerobic fitness (estimation of VO_2max_), and calculates a total fitness score [18]. In addition, the MicroFit data contained the name, age, sex, and the date the student took the test. For statistical analysis the names of the students were removed. For the testing we used the MicroFit FAS-2 System which consist of automated system for measuring resting blood pressure and heart rate, interactive skinfold calliper, flexometer for flexibility testing, medical scale for measuring weight and biceps strength, a stationary bike for cardiovascular fitness testing, chest strap transmitter and heart rate receiving unit and the *MicroFit Health Wizard* software version 5.3.5. 

### 2.3. Body composition

Body composition evaluation measured the major structural components of the body: muscles, and fat. The height was measured in cm and the weight in kg. Body Mass Index (BMI) was calculated using the weight and height of the student (BMI = W/H², where W is weight in kilograms and H^2^ is height in meters squared). The skinfold test, used to calculate the percent body fat, was measured in millimeters and taken from three sites on the right side of the body: chest, abdomen, and thigh for males, and triceps, suprailium and thigh for females. For males, the three skinfold measurements were added up (MSF = chest + abdomen + thigh) and computed with the Jackson-Pollock method [19] to measure body density: Db = 1.10938 − 0.0008267(MSF) + 0.0000016(MSF)^2^ − 0.0002574(age). The following formula was then used to compute % body fat: % Body Fat = [(4.95/Db) − 4.5] × 100. For females, the three skinfold measurements were added up (FSF = triceps + suprailium + thigh) and computed with the Jackson-Pollock-Ward method [20] to measure body density: Db = 1.10994921 − 0.0009929(FSF) + 0.0000023(FSF)^2^ − 0.0001392(age). The following formula was then used to compute % body fat: % Body Fat = [(5.01/Db) − 4.57] × 100. All calculations were done automatically by the computer.

### 2.4. Blood pressure and resting heart rate

Blood pressure was measured using the oscillometric method with an automated MicroFit FAS-2 blood pressure system. Blood pressure was measured before any active test like biceps strength or aerobic fitness. The participant was asked to sit quietly for 5 minutes before the measurement. During the measurement the participant was sitting upright, relaxed with both feet flat on the floor, and their arm resting on the table. The cuff was wrapped around their upper arm so the bottom edge of the cuff was 2–3 cm above the point where the upper arm joins the lower arm. The lower edge of the cuff was at the level of the participant’s heart. The blood pressure cuff was connected to the computer and the measurement was automatic. The systolic and diastolic blood pressure was reported in millimeters of mercury (mmHg). 

Resting heart rate was determined automatically at the end of the blood pressure measurement. If the participants resting heart rate was above 100 beats per minute (bpm) he/she was asked to wait another 5 minutes to repeat the measurement. If the heart rate remained high the participant was told that their resting heart rate was out of range for the Fitness Profile, and that he/she should mention the elevated heart rate during their next visit with their physician.

### 2.5. Muscle strength

The biceps strength test measures the maximum force generated in a single pull. This is a static test where the elbows are fixed at 90 degrees and there is no bar movement during the pull. This measurement was done using the static strength-testing device the MicroFit FAS-2 strength platform. Before the test the technician asked the participant: “Do you have a back or arm injury or is there any other reason you should not lift heavy objects?” If the participant’s answer was positive, the biceps test was skipped. The participants were instructed to keep their back straight during the pull and to rotate their pelvis forward by squeezing their buttocks together. Holding the bar with palms facing up and the strap adjusted so the elbow join was at 90 degrees, the participants were asked to pull the bar up using biceps muscles only for 3 seconds. The program then calculated the final strength score in kilograms.

### 2.6. Flexibility

Flexibility was assessed using the MicroFit FAS-2 flexometer to measure lower back and hamstring flexibility. The participants sat on the floor, with their shoes off, their legs straight, and feet against the flexometer foot stop. Before the test the technician asked the participant: “Do you have a back injury or is there any other reason you should not try to touch your toes?” If the participant’s answer was positive, the flexibility test was skipped. When participant reached forward and touched the flexometer for 3 seconds, a measurement was recorded in centimeters.

### 2.7. VO_2max_—the maximum uptake of oxygen during a graded exercise

Estimation of VO_2max_ measured the maximum uptake of oxygen during a graded exercise. This study estimated the VO_2max_ using the Åstrand bike protocol. This protocol was recommended for young and middle-age adults. The Åstrand bike protocol started with a three minute work stage and then used three additional two minutes work stages thereafter. If the heart rate at the end of a work stage was below the Threshold Exercise Heart Rate (TEHR), the work load depending on the heart rate was increased by 0.5 kp (25 Watt) or 1.0 kp (50 Watt). If heart rate at the end of a work stage was above the TEHR, the work level was maintained for three more minutes and then the test was complete. The TEHR for ages below 50 years is 120 bpm, for ages above 50 years is 125 bpm. The VO_2max_ was then calculated based upon the final work load and the average of the last two minute heart rates. The formula for VO_2max_ estimation was: VO_2max_ (mL/kg/min) = *VO_2max_ (from table)* × 1000/weight (kg) × *age factor* [21]. The age factor was computed using the following equation: 100/(1.37 × age + 66.8). Before the test, male participants answered the following question: “During the past three months did you engage in vigorous activities like running or cycling for at least 15 minutes/day on three days/week?” If the answer was positive, the initial load was set automatically at 2.0 kp (100 Watt). If the answer was negative the initial load was 1.5 kp (75 Watt). For females the initial load was constantly set at 1.0 kp (50 Watt). The participants wore a heart rate monitor during the test. The computer responded to changes in heart rate by automatically adjusting the work load level of the bicycle ergometer. The entire test takes ten minutes, including a two-minute cool down period at the end. The computer then produces a printout with the test results and comparison of the results to the national norms for the appropriate age and sex group. 

### 2.8. Statistical analysis

The Statistical Package for the Social Sciences (SPSS; version 18.0) was used for the data analysis. A linear regression was used to measure trends between VO_2max,_ BMI, body composition, and years. Pearson correlations were computed between VO_2max,_ BMI and body composition. *P* values less than or equal to 0.05 on two-sided tests were considered statistically significant.

## 3. Results and Discussion

### 3.1. Demographics

Table 1 describes the characteristics of the study population. Out of the 5101 Andrews University students who participated in this study, 45% were males and 55% were females. Mean % body fat for males was 11.6% and 22.4% for the females, respectively. The mean VO_2max_ for males was 38.7 mL/kg/min and 34.2 mL/kg/min for females, respectively. The mean BMI was 24.1 kg/m^2^ for males and 24.0 kg/m^2^ for females, respectively.

**Table 1:** Characteristics of the study population.

	Males	Females
Gender (%, n)	44.5 (2273)	55.4 (2828)
Age (years; mean ± SD)	21.5 ± 4.6	21.9 ± 5.8
% Body Fat (mean ± SD)	11.6 ± 6.5	22.4 ± 6.7
Bicep Strength (kg; mean ± SD)	41.7 ± 12.0	23.8 ± 7.4
Flexibility (cm; mean ± SD)	37.8 ± 12.1	43.1 ± 11.6
Systolic BP (mmHg; mean ± SD)	129.4 ± 15.3	118.4 ± 14.1
Diastolic BP (mmHg; mean ± SD)	76.7 ± 10.2	73.5 ± 9.4
Resting Heart Rate (bpm; mean ± SD)	73.1 ± 13.5	78.7 ± 12.9
VO_2max_ (mL/kg/min; mean ± SD)	38.7 ± 11.0	34.2 ± 10.2
Height (cm; mean ± SD)	175.0 ± 7.5	162.2 ± 7.0
Weight (kg; mean ± SD)	77.1 ± 15.8	65.6 ± 16.0
BMI (kg/m^2^; mean ± SD)	24.1 ± 4.5	24.0 ± 5.3
BMI: body mass index; BP: blood pressure; SD: standard deviation.		

### 3.2. Trends in fitness levels

**Figure 1:**
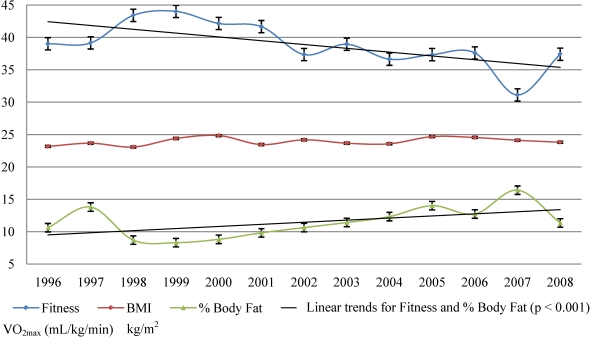
Trends in physical fitness, BMI and % body fat in males (1996–2008).

**Figure 2:**
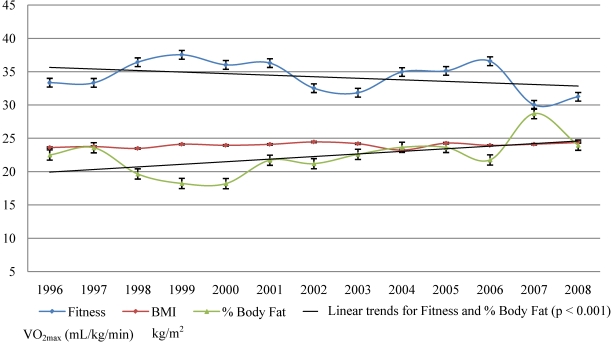
Trends in physical fitness, BMI and % body fat in females (1996–2008).

Figure 1 and Figure 2 represents the trends in physical fitness as expressed in estimates of VO_2max_ and BMI and % body fat for males and females between the years 1996 and 2008. There is a significant linear trend between the VO_2max_ and the years (r = −0.248, p < 0.001) for males as well for females (r = −0.135, p < 0.001). On average, in the last 13 years the VO_2max_ was decreasing 0.812 mL/kg/min a year for males and 0.414 mL/kg/min a year for females.

There was a noticeable increase in VO_2max_ in 1998 and 1999, followed by noticeable decreases in VO_2max_ in males and females in the years 2000, 2002 and 2007. Females step by step recovered back between 2003 and 2006. In the year 2007 we observed the largest decline in VO_2max_ in males and females out of the last 13 years. In 2008, the level of physical activity for the male population bounced back, with an increase in VO_2max_, while the female results showed less improvement.

### 3.3. Trends in BMI

The linear trend for BMI by year was not significant for both sexes (for males p = 0.772, p for females = 0.253). Combining data for all years there was a significant indirect correlation between the student’s VO_2max_ levels and BMI, r = −0.334; p < 0.001 for males; and r = −0.414, p < 0.001 for females. Approximately 11.1% of the variance in the VO_2max_ levels in males and 17.1% in females can be explained by the variance in BMI.

### 3.4. Trends in % body fat

One of the problems associated with BMI is that there is no way to know if the person is heavily muscled or overweight. Therefore, body fat percentage has been recently recommended as a more accurate measurement of body fatness [22,23,24,25]. There is a significant linear trend between the % body fat and the years (r = 0.264, p < 0.001) for males as well as for females (r = 0.324, p < 0.001). On average, in the last 13 years, % body fat increased 0.513%/year for males and 0.654%/year for females. There is a significant indirect correlation between the student’s VO_2max_ levels and % body fat, r = −0.489; p < 0.001 for males; and r = −0.416, p < 0.001 for females. Approximately 23.9% of the variance in the VO_2max_ levels in males and 17.3% in females can be explained by the variance in % body fat.

### 3.5. Fitness categories

**Figure 3:**
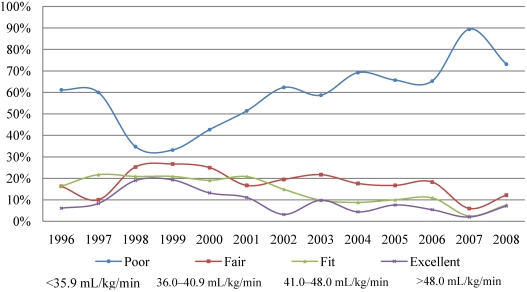
Percentage of males falling into the four difference fitness categories (1996–2008).

**Figure 4:**
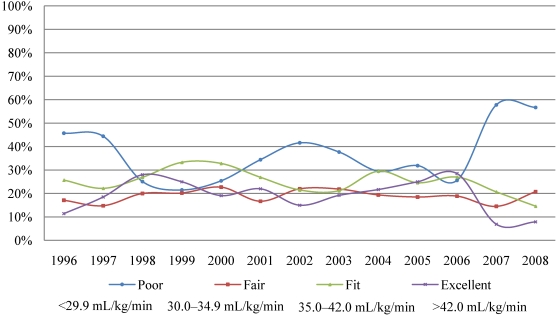
Percentage of females falling into the four difference fitness categories (1996–2008).

Using MicroFit’s own criteria for appropriate age and sex we have divided the students according to their aerobic fitness VO_2max_ results into four fitness categories—poor, fair, fit and excellent. Figure 3 and Figure 4 show the percentage of males and females who fell into these four fitness categories. The decline in physical fitness occurred differently in males and females. Starting with the year 1999, males dramatically increased in the poor category. The trend peaked in the year 2007 and there was a rebound in the year 2008. While the poor category increased disproportionally, there were fewer and fewer males, who could be considered excellent, fit or fair (Figure 3). In contrast to males, the percentage of females in the poor category fluctuated up and down. There were several rebounds, in 2003, 2006 and 2008; however, they were not as pronounced as in males. In 2007 there was a dramatic increase of females in the poor category. As the poor fitness category increased there were fewer losses from the fair, fit, and excellent categories in females in comparison to males (Figure 4). Although both sexes have declined in their physical fitness levels, the results from our study show that there is a more pronounced and deeper decline in the males then females.

The major finding of this study was the slow, gradual decline in fitness levels during the last 13 years, measured as an estimation of maximal oxygen consumption (VO_2max_) on a bicycle ergometer in college men and women. At the same time, we observed a slow increase in % body fat values for both sexes. The value of our study is the reported estimation of VO_2max_, which is the most accurate measurement of physical fitness. In the last few years, several publications have reported indirect indications of physical fitness, like steps walked per day, or time spent participating in physical activities [12,13,26,27,28].

The Dietary Guidelines for Americans [29] indicate that adults should perform moderate activity for at least 30 minutes daily, or most of the week, and participate in activities to strengthen muscles and improve flexibility. The 2008 Physical Activity Guidelines for Americans [30] recommend that adolescents should engage in 60 minutes or more of physical activity daily. Most of the 60 minutes or more should be either moderate- or vigorous-intensity aerobic physical activity, and should include vigorous-intensity physical activity at least three days a week. As part of their 60 or more minutes of daily physical activity, children and adolescent should include muscle-strengthening physical activity and bone-strengthening physical activity on at least three days of the week. Students who follow these guidelines should be able to improve or at least maintain their VO_2max_ levels. Unfortunately, research indicates that college years are characterized by the worsening of food habits [31], meal skipping [32,33], snacking [34], and frequent consumption of fast foods [32,34]. College years are also characterized by rapid reduction in physical activity [1,2,12,35] and the beginning of a sedentary lifestyle [36].

### 3.6. Limitations

There are a few limitations to the study that need to be considered. First, the technicians who collected the data changed over the time period in which students were taking the MicroFit test, and therefore the techniques between technicians may have varied. Secondly, the software and testing equipment experienced difficulty at random times which contributed to some missing data.

The students took the exam at different times throughout the semester as part of their physical education activity course requirements. Students who took the MicroFit test at the beginning of the semester may have had worse results that those who took the test later in the semester after participating in a physical activity. However, there is no evidence that only students with a poor fitness score completed the MicroFit at the beginning of the semester and thus it can be assumed that the fitness scores averaged over the course of the school semester. Because MicroFit does not contain data on ethnicity, generalizability of the results may be limited.

## 4. Conclusions

Our findings show that there is a small and declining minority of male and female college students who are physically in shape. The MicroFit data shows that the fitness levels measured as estimation of VO_2max_ have gradually decreased among males and females over the past 13 years. The BMI and % body fat has fluctuated up and down, and is significantly indirectly correlated with VO_2max_ levels. As VO_2max_ decreased, the BMI and % body fat increased for both sexes. The observed trends are unfortunate because it has been demonstrated that physical activity and good nutrition can have a positive effect on the overall performance of students. Physical activity can reduce stress levels and improve work-related time management. Strategies should be implemented to counter the present trend and help young people improve their physical fitness.

## References

[1] American College Health Association (2006). American College Health Association-National College Health Assessment (ACHA-NCHA) Spring 2005 Reference Group Data Report (Abridged). J. Am. Coll. Health.

[2] American College Health Association National College Health Assessment Reference Group Executive Summary Fall 2008. http://www.acha-ncha.org/docs/ACHA-NCHA_Reference_Group_ExecutiveSummary_Fall2008.pdf.

[3] Sacheck J.M., Kuder J.F., Economos C.D. (2010). Physical fitness, adiposity, and metabolic risk factors in young college students. Med. Sci. Sports Exerc..

[4] Powell K.E., Dishman R.K. (1988). Habitual exercise and public health: An epidemiological view. Exercise Adherence: Its Impact on Public Health.

[5] Shaw K., Gennat H., O’Rourke P., Del Mar C. (2006). Exercise for overweight or obesity. Cochrane Database Syst. Rev..

[6] Coyle Y.M. (2009). Lifestyle, genes, and cancer. Methods Mol. Biol..

[7] Brown J.D. (1991). Staying fit and staying well: physical fitness as moderator of life stress. J. Pers. Soc. Psychol..

[8] Pertruzello S.J., Landers D.M., Hatfield B.D., Kubitz K.A., Salazar W. (1991). A meta-analysis on the anxiety-reducing effects of acute and chronic exercise. Outcomes and mechanism. Sports Med..

[9] Crews D.J., Landers D.M. (1987). A meta-analytic review of aerobic fitness and reactivity to psychological stressors. Med. Sci. Sports Exerc..

[10] Etnier J.L., Salazar W., Landers D.M., Petruzzelo S.J., Myungwoo H., Nowell P. (1997). The influence of physical fitness and exercise upon cognitive functioning: a meta-analysis. J. Sport Exerc. Psychol..

[11] Douglas K.A., Collin J.L., Warren C., Kann L., Gold R., Clayton S., Ross J.G., Kolbe L.J. (1997). Results from the 1995 national health risk behaviour survey. J. Am. Coll. Health.

[12] Bray S.R., Born H.A. (2004). Transition to university and vigorous physical activity: implications for health and psychological well-being during transition to university life. J. Am. Coll. Health.

[13] Lowry R., Galuska D.A., Fluton J.E., Wechsler H., Kann L., Collins J.L. (2008). Physical activity, food, choice, and weight management goals and practices among US college students. Am. J. Prev. Med..

[14] Center for Disease Control The Behavioral Risk Factor Surveillance System (BRFSS). http://www.cdc.gov/brfss/index.htm.

[15] Beeson L.W., Mills P.K., Phillips R.L., Andress M., Fraser G.E. (1989). Chronic disease among Seventh-day Adventists, a low risk group. Cancer.

[16] Fraser G.E., Shavlik D.J. (2001). Ten Years of life—Is it a matter of choice?. Arch. Intern. Med..

[17] White E.G. (1905). The Ministry of Healing.

[18] MicroFit Health and Fitness Systems. Colleges & Universities.

[19] Jackson A.S., Pollock M.L. (1978). Generalized equations for prediction body density of men. Br. J. Nutr..

[20] Jackson A.S., Pollock M.L., Ward A. (1980). Generalized equations for predicting body density of women. Med. Sci. Sports Exerc..

[21] Åstrand P.-O., Ryhming I. (1954). A nomogram for calculation of aerobic capacity (physical fitness) from pulse rate during submaximal work. J. Appl. Physiol..

[22] Wang J., Thornton J.C., Kolesnik S., Pierson R.N. (2000). Anthropometry in body composition. An overview. Ann. N. Y. Acad. Sci..

[23] Powers S.K., Howley E.T. (2009). Exercise Physiology: Theory and Application to Fitness and Performance.

[24] McArdle W.D., Katch F.I., Katch V.L. (1991). Exercise Physiology: Energy, Nutrition, and Human Performance.

[25] Wilmore J.H., Costill D.L. (2004). Physiology of Sport and Exercise.

[26] Mestek M.L., Paisance E., Grandjean P. (2008). The relationship between pedometer-determined and self-reported physical activity and body composition variables in college-aged men and women. J. Am. Coll. Health.

[27] McArthur L.H., Raedeke T.D. (2009). Race and sex in college students physical activity correlates. Am. J. Health Behav..

[28] Taliaferro L.A., Rienzo B.A., Pigg R.M., Miller M.D., Dodd V.J. (2008). Association between physical activity and reduced rates of hopelessness, depression, and suicidal behavior among college students. J. Am. Coll. Health.

[29] USDA/HHS (2000). Nutrition and Your Health: Dietary Guidelines for Americans.

[30] USDA/HHS (2008). 2008 Physical Activity Guidelines for Americans.

[31] Grace T.W. (1997). Health problems of college students. J. Am. Coll. Health.

[32] Nicklas T.A., Baranowski T., Cullen K.W., Bergerson G. (2001). Eating patterns, dietary quality and obesity. J. Am. Coll. Health.

[33] Center for Disease Control and Prevention; National Center for Chronic Disease Prevention and Health Promotion (1996). Physical Activity and Health: A Report of the Surgeon General.

[34] Hertzler A.A., Webb R., Frary R. (1995). Overconsumption of fat by college students, the fast food connection. Ecol. Food Nutr..

[35] Grubbs L., Carter J. (2002). The relationship of perceived benefits and barriers to reported exercise behaviours in college undergraduates. Fam. Community Health.

[36] Pinto B.M., Marcus B.H. (1995). A stage of change approach to understanding college students’ physical activity. J. Am. Coll. Health.

